# 1189. Clonal Recovery Pattern of *Staphylococcus aureus* during Sham-controlled, Interventional, Crossover Trial on the Effectiveness of Pulsed Xenon Ultraviolet Light (PX-UV) in Reducing Healthcare-associated Infections

**DOI:** 10.1093/ofid/ofac492.1024

**Published:** 2022-12-15

**Authors:** Thanuri Navarathna, Piyali Chatterjee, Landon Ashby, Hosoon Choi, Munok Hwang, Sorabh Dhar, Keith S Kaye, Chetan Jinadatha

**Affiliations:** Central Texas Veterans Health Care System, Temple, Texas; Central Texas Veterans Health Care System, Temple, Texas; Central Texas Veterans Health Care System, Temple, Texas; Central Texas Veterans Health Care System, Temple, Texas; Central Texas Veterans Health Care System, Temple, Texas; Wayne State University, Detroit, Michigan; Rutgers - Robert Wood Johnson Medical School, New Brunswick, New Jersey; Central Texas Veterans Health Care System, Temple, Texas

## Abstract

**Background:**

PX-UV has shown great effectiveness at reducing *Staphylococcus aureus* (*S. aureus*) on healthcare surfaces. *S. aureus* is one of the most prevalent causes of healthcare-associated infections (HAIs). Here we assess the clonal recovery pattern and the disruption caused by PX-UV on *S. aureus* collected from patients, using Whole Genome Multilocus Sequence Type (wgMLST) analysis.

**Methods:**

During a 3-year period, 2017 to 2020, a prospective sham-controlled double-blinded interventional crossover trial designed to assess the effectiveness of post-discharge PX-UV room disinfection on HAIs in 2 Detroit hospitals. The interventional group (Q) and the control group (W) was treated with PX-UV and sham UV light after terminal cleaning respectively, both treatments lasting 12 months. Crossover occurred after a 6-month washout period (R) during which no UV light was used. Whole Genome Sequencing (WGS) of the 291 *S. aureus* samples collected was performed using the Nextseq 550 (Illumina). Following *de novo* assembly, construction of minimum spanning tree (MST) and wgMLST analysis was completed using BioNumerics calculation engine (v7.6).

**Results:**

A total of 20 sequence types (STs) were found throughout the trial, the most common being ST5 and ST8 with roughly 20 to 50 each found in all 3 treatment groups (Table 1. The other 18 STs were only found 7 times or less in each treatment group. ). During intervention (Q) 9 STs were found, however 11 were not recovered after PX-UV (ST1, ST1508, ST22, ST231, ST3287, ST3462, ST398, ST59, ST6790, ST840, ST97). During the sham treatment (W) 15 STs were found while 7 were found during the washout period (R). The minimum spanning tree (MST) shows clusters of related ST groups color coded by treatment type (Figure 1).

**Fig. 1:**
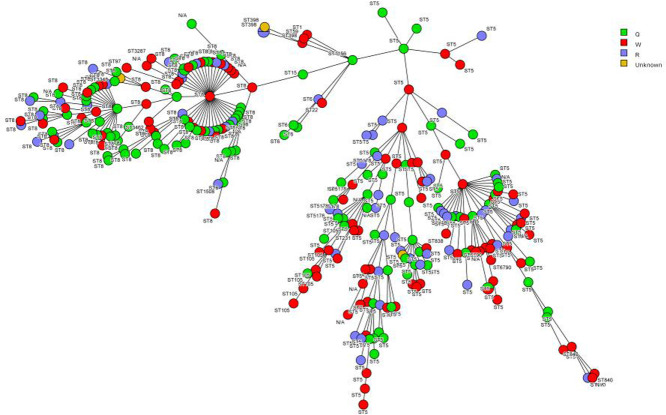
Minimum spanning tree (MST) for S. aureus

**Table 1:**
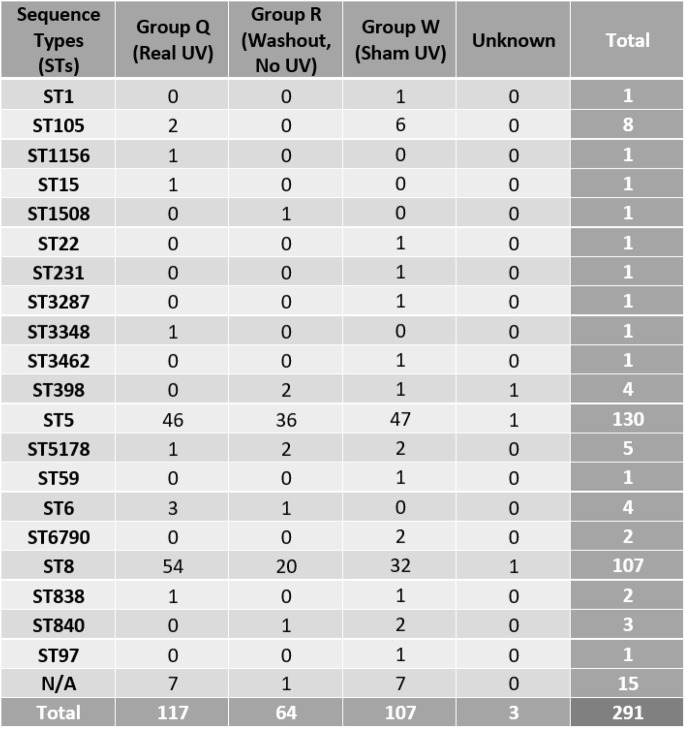
Total number of each sequence type (ST) per treatment group

**Conclusion:**

The difference between the STs recovered during intervention and during sham UV treatment was inconsequential because many factors influence the amount of STs recovered (treatment, number of patients, sampling bias, and terminal cleaning methods). There was no clonality noted among any of the intervention group (Fig. 1). Both dominant strains (ST5, ST8) have been characterized as hypervirulent and predominant previously, consistent with our data. Our data does not demonstrate any notable change in the clonal prevalence of *S. aureus* after PX-UV use.

**Disclosures:**

**Piyali Chatterjee, PhD**, AHRQ Grant # 1R03HS027667-01: Grant/Research Support|AHRQ Grant # 1R03HS027667-01: Central Texas Veterans Health Care System **Keith S. Kaye, MD, MPH**, Allecra: Advisor/Consultant|GlaxoSmithKline plc.: Receiving symposia honoraria|GlaxoSmithKline plc.: GlaxoSmithKline plc.-sponsored study 212502|Merck: Advisor/Consultant|qpex: Advisor/Consultant|Shionogi: Grant/Research Support|Spero: Advisor/Consultant **Chetan Jinadatha, MD, MPH**, AHRQ R01 Grant-5R01HS025598: Grant/Research Support|EOS Surfaces: Copper Coupons and materials for testing.

